# Nutraceutical Boom in Cancer: Inside the Labyrinth of Reactive Oxygen Species

**DOI:** 10.3390/ijms21061936

**Published:** 2020-03-12

**Authors:** Maura Calvani, Amada Pasha, Claudio Favre

**Affiliations:** 1 Division of Pediatric Oncology/Hematology, Meyer University Children’s Hospital, 50139 Florence, Italy; maura.calvani@meyer.it (M.C.); claudio.favre@meyer.it (C.F.); 2 Department of Health Sciences, University of Florence, 50139 Florence, Italy

**Keywords:** nutraceuticals, cancer, phenolic compounds

## Abstract

In recent years, epidemiological studies have shown that food is a very powerful means for maintaining a state of well-being and for health prevention. Many degenerative, autoimmune and neoplastic diseases are related to nutrition and the nutrient–organism interaction could define the balance between health and disease. Nutrients and dietary components influence epigenetic phenomena and modify drugs response; therefore, these food–host interactions can influence the individual predisposition to disease and its potential therapeutic response. Do nutraceuticals have positive or negative effects during chemotherapy? The use of nutraceutical supplements in cancer patients is a controversial debate without a definitive conclusion to date. During cancer treatment, patients take nutraceuticals to alleviate drug toxicity and improve long-term results. Some nutraceuticals may potentiate the effect of cytotoxic chemotherapy by inducing cell growth arrest, cell differentiation, and alteration of the redox state of cells, but in some cases, high levels of them may interfere with the effectiveness of chemotherapy, making cancer cells less reactive to chemotherapy. In this review, we highlighted the emerging opinions and data on the pros and cons on the use of nutraceutical supplements during chemotherapy.

## 1. Introduction

### 1.1. Nutraceuticals

In 1989 Stephen DeFelice coined for the first time the neologism “Nutraceuticals”, which derives from words “nutrition” and “pharmaceutical”, describing this term as “the food components or active ingredients present in food that have positive effects for well-being and health, including the prevention and treatment of diseases” [1]. Prior to the 1990s the term nutraceutical was not in use, but there was research on the functional properties and ability of food to alleviate and/or prevent certain conditions connected to different type of diseases. According to DeFelice, nutraceuticals include foodstuffs, dietary supplements and medical foods, with a distinctive health impact in either prevention and/or treatment of diseases [1]. An emerging area of research comprehends the use of nutraceuticals in the prevention and management of chronic diseases, but to confirm their disease-related benefits, a longer-term clinical trial is needed [2].

Nutraceuticals are food constituents, extracts, or food derivatives such as vitamins, amino acids, minerals that can have potential health benefits beyond their nutritional value [3]; they are referred to as dietary supplements, and from a nutritional perspective they are a source of both nutrients (carbohydrates, proteins, fats) and non-nutrients (e.g., prebiotics, probiotics, phytochemicals, enzymatic regulators) [2]. In recent years, numerous scientific studies have shown that food is a very powerful means at our disposal for safeguarding the body in maintaining a state of well-being [3]. In fact, many degenerative, autoimmune and neoplastic diseases are positively and negatively related to nutrition, and the nutrient–organism interaction could define, throughout life, the balance between health and disease [1]. Several in vitro and in vivo studies suggest that nutraceuticals have a protective effect against chronic diseases, but the results are not supported fully by clinical evidence [4]. It has also been demonstrated that they may serve as a useful adjunct to pharmaceuticals to better manage chronic conditions or offset negative side effects. In the 19th century, in line with Hippocrates’ philosophy “let the food be thy medicine and thy medicine be the food” the connection between nutrition and human health was conceptualized, as well as the relationship between the use of appropriate foods for health and their therapeutic benefits.

In the literature there is an abundance of terminology, definitions and designations of nutraceuticals that lead to confusion. To be considered “functional food”, food must comply with three specific conditions: (1) be part of a normal daily diet; (2) be a natural ingredient, not occurring in the form of pills, capsules, or any medical/pharmacological form; and (3) once consumed, it has to improve/regulate a specific metabolic process/mechanism, in that way preventing or controlling a disease [5].

Bioactive phytochemicals like alkaloids, various terpenoids, and polyphenols (anthocyanins, flavones, flavanols, isoflavones, stilbenes, ellagic acid, etc.) are a significant source for nutraceutical ingredients [6]. Phytochemicals produced mainly by plants, are non-essential nutrients that have either defensive or disease protective properties [7]; in human health they play specific pharmacological effects as antioxidants, anti-inflammatory, anti-allergic, antibacterial, antifungal, chemo-preventive, hepato-protective, neuroprotective, hypotensive, antiaging in diabetes, osteoporosis, DNA damage, cancer and heart diseases [6,7].

### 1.2. Nutraceuticals and Cancer

Epidemiological studies have consistently shown that dietary habit is one of the most important determinants of chronic diseases such as cardiovascular diseases, type-II diabetes, gallstone, neurodegenerative diseases, cataract and several types of cancer (e.g., gastrointestinal cancer). Food and dietary habits have a direct impact on health and diseases [8]. Biochemical and epidemiological evidence has shown that humans have evolved a sophisticated and co-operative system of antioxidants defense mechanism against toxic oxygen intermediates through nutrition. The increased intake of antioxidants through nutrition may lower the risk of diseases such as cancer [9]. Nutraceuticals, functional foods and supplemental micronutrients have the potential to reduce cancer cell growth, inhibiting cell proliferation and inducing cancer cells apoptosis [10]. A bunch of natural dietary products have shown a potential role in prevention and treatment of cancers and have been considered an effective strategy [11]. Natural and dietary nutraceuticals may possess anticancer activities, like carotenoids that promote gap-junctional communication, in vitro, through amplification of “connexin 43”, [12] or flavonoids, which modulate phase I and II xenobiotic detoxification, or vitamin E that inhibits protein kinase C, a critical enzyme in tumor progression of some types of cancer [13]. Curcumin, Resveratrol (RES) and Berberine (BBR) have been examined in 126, 110 and 35 clinical trials respectively and have shown many different activities in human pathologies such as cardiovascular diseases, colorectal adenoma reoccurrence, diabetes, defective endothelial function, glucose metabolism/metabolic syndrome, age-related macular degeneration, aging, Alzheimer’s disease, different types of cancer and many other pathologies [14]. Many reports state that phytochemical compounds (e.g., from cumin, red pepper and ginger) can potentially prevent cancer by suppressing the pathway of Nuclear transcription factor-κB (NF-κB) correlated with cancer and many inflammatory diseases. NF-κB is a very attractive therapeutic target for plant-derived nutraceuticals and polyphenols [15]. It has been reported that NF-κB activity is altered also by RES which can inhibit cytochrome P450 isoenzyme (CYPA1) drug metabolism and cyclooxygenase activity. Moreover, RES may influence other biochemical pathways related to metabolism, fatty acids oxidation, mitochondrial biogenesis and respiration and gluconeogenesis [16]. RES may induce apoptosis of activated T cells and suppress tumor necrosis factor-alpha (TNF-α), interleukin 17 (IL-17) and additional pro-inflammatory cytokines. Hence, it has been proposed that it may be useful in auto-immune diseases [17]. Curcumin and its analogs have attracted great attention as cancer-preventive agents through their anti-cancer activities including inhibition of cell proliferation, anti-invasive activity, and inhibition of angiogenesis in vitro [18]. Curcumin inhibits mouse liver lymphoma through activating Nrf2 enzymes, promoting tumor suppressor p53 and reducing TGF-β and COX-2 [19]. Many dietary natural products could affect the development and progression of breast cancer (BC), such as soy, pomegranate, mangosteen, citrus fruits, apple, grape, mango, cruciferous vegetables, ginger, garlic, black cumin, edible macro-fungi, and cereals. Their anti-breast cancer effects involve various mechanisms of action, such as downregulating ER-α expression and activity, inhibiting proliferation, migration, metastasis and angiogenesis of breast tumor cells, inducing apoptosis and cell cycle arrest, and sensitizing breast tumor cells to radiotherapy and chemotherapy [20].

Between 20% and 85% of patients use dietary supplements after being diagnosed with cancer [21]. Supplements are most used by BC survivors, by patients with prostate, colorectal and lung cancer, since these are the most common types of cancer diagnosed in adults [22]. The use of dietary supplements is highly debated, particularly when patients are undergoing treatment because it is unknown whether supplements affect treatment efficacy.

Do nutraceutical dietary supplements give benefits in patients affected by chronic diseases or not? Are there any benefits in the dietary supplements commonly used during chemotherapies for their antioxidant and pro-oxidant properties?

## 2. Oxidative Stress (OS)

**Figure 1:**
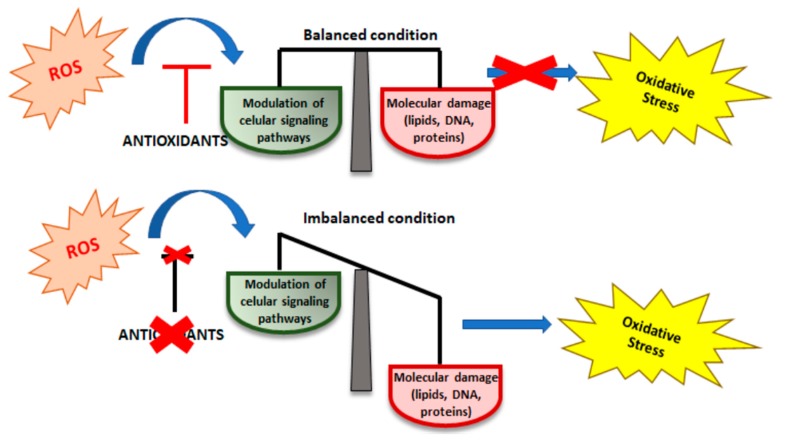
Balance between ROS production and antioxidant defense.

An appropriate equilibrium between oxidation and antioxidants is fundamental to life. A disruption in the pro-oxidant-antioxidant balance leads to “oxidative stress” (OS), which may induce tissue injury [23]. OS is a physiological process that is generated in the body when there is an increase in oxidant processes due to a lack of antioxidant defense. This imbalance can activate different cell pathways involved in cancer progression; if OS becomes excessive or a permanent condition, it may determine a disease [24]. OS can damage all the constituents of the body (proteins, lipids, fatty acids, DNA, etc.) [25]. Pathologies such as cardiovascular diseases, cancer, atherosclerosis, diabetes [26,27], and neurological and endocrinological disorders have been related to upregulation of OS, due to an excessive production of reactive oxygen species (ROS) or to a decreased scavenging contribution (Figure 1) [24]. The uncontrolled increase of ROS has been considered a common pathway for the initiation and progression of numerous diseases including cancer. Indeed, altered redox balance and deregulated redox signaling are two common hallmarks of tumors that are strongly implicated in malignant progression and resistance to treatment [28]. Reactive species or free radicals include reactive oxygen and nitrogen species collectively and are termed reactive oxygen nitrogen species (RONS) [29]. In addition to ROS, reactive nitrogen species (RNS) are generated from physiological processes to produce metabolites and energy as a defense against invasive microorganisms [30]. Low levels of ROS are essential for normal physiological functions of the cell as they promote mitogenic proliferation and act as crucial second messengers in many redox-sensitive signaling cascades [31]. Moderate amounts of ROS have positive effects in wound healing, repairing processes and against invading pathogens [32]. Tumor cells regulate multiple enzymes and use their metabolic pathways to provide an adequate supply of antioxidant systems (antioxidant enzymes in conjunction with non-enzymatic antioxidants such as glutathione, thioredoxin, vitamins A, C and E). Cells’ antioxidant ability represents a strong foundation to counteract the damage of OS [33]. Cells develop various sophisticated mechanisms for maintaining redox homeostasis in order to neutralize the deleterious effects of ROS. Antioxidants could be classified into two types: direct antioxidants that have redox activity and short half-lives that should be supplemented or regenerated during the process [34] and indirect antioxidants, whose physiological effects last longer, act through the augmentation of cellular antioxidant capacity by enhancing the expression of specific genes, such as *NFE2L2* encoding for the nuclear factor (erythroid-derived 2)-like 2 (Nrf2), known as a master regulator of the antioxidant response [35,36]. Furthermore, indirect antioxidants are unlikely to evoke pro-oxidant effects which have been a problem in the use of high dose vitamin E therapy [36].

### 2.1. Antioxidant Activity of Nutraceuticals

The Panel on Dietary Antioxidants and Related Compounds of the Food and Nutrition Boards defines that “a dietary antioxidant is a substance in food that significantly decreases the adverse effects of ROS, RNS or both on normal physiological function in humans” [37].

Free radicals are involved in the multistage carcinogenic process. Peroxyl radicals and lipid peroxidation can cause mutations on DNA, which are crucial for the initiation of the tumorigenic process [38]. Antioxidant phytochemicals may modulate the initiation of carcinogenesis by protecting against DNA damage. Antioxidants counteract free radicals and neutralize oxidants using a system consisting of enzymatic antioxidants such as superoxide dismutase (SOD), catalase (CAT), glutathione peroxidase (GPx) and thioredoxin (Trx), as well as the non-enzymatic antioxidants [36]. Antioxidants can be classified according to their source and include endogenous synthesis such as enzymes and small molecules, as well as exogenous diets such as phenolics, flavonoids, phenolic acids, carotenoids, vitamins and minerals [39]. Some natural antioxidants as carotenoids and polyphenols can prevent many diseases by reducing OS [35,40]. Phytochemicals, plant-derived non-nutritive compounds, and many natural compounds present in food are of great interest due to their antioxidant capacities and their beneficial effects on human health [41]. Many dietary phytochemicals derived from various vegetables, fruits, spices and herbal medicines can activate Nrf2 and induce expression of antioxidant or phase II detoxifying enzymes in vitro [38,42]. Epidemiological and animal studies suggest that the regular consumption of fruits, vegetables and whole grains reduces the risk of chronic diseases associated with oxidative damage [43]. Another important factor that may cause or promote the pathogenesis of many chronic diseases, including cancers and type 2 diabetes, is chronic inflammation [44] and most antioxidant phytochemicals have been found to have anti-inflammatory properties.

Resveratrol, curcumin and anthocyanins, a class of phytochemicals, can reduce inflammation inhibiting prostaglandin production and NF-κB activity, enzyme activity, as well as increase of cytokine production [45]. Flavonoids appear to reduce the risk of death from coronary heart disease [46,47]. Elevated levels of ROS have been detected in almost all cancers, where they promote many aspects of tumor development and progression [31]. Many bioactive compounds are demonstrated to inhibit different pathways involved in angiogenesis, tumorigenesis and metastasis in pediatric and adult tumors disrupting different cellular enzymes such as DNA telomerase and topoisomerase [48].

Catechins are monomers of flavanols with a variety of similar compounds comprising catechin, epigallocatechin, epicatechin gallate (EGC) and epigallocatechin gallate (EGCG), which is the major component of polyphenols found in green tea [49]. Studies in animal models of carcinogenesis have shown that green tea and EGCG can inhibit cancer cell proliferation. EGCG, curcumin and resveratrol can block NF-kB activation thus inhibiting the expression of COX-2 and nitric oxide synthase (NOS) [50].

Berberine (BBR) is another anti-cancer drug extracted from traditional Chinese herbal medicines that can bind to oligonucleotides to stabilize DNA triplexes or G-quadruplexes and can reduce the growth of many kind of tumors inhibiting telomerase and topoisomerase [51,52]. Data have shown that BBR can arrest growth, carcinogenesis and metastasis in almost all types of cancers, as it has a powerful effect on crucial cancer pathways such as the mitogen-activated protein kinase (MAPK) and the NF-κB pathway [53].

Lycopene is a non-provitamin A carotenoid powerful antioxidant that is abundant in tomato and its derivatives and provides protection against cell damage due to free radicals [54,55]. Linnewiel et al. observed that the supplementation of lycopene in rats increased the expression of Nrf2, HO-1, glutathione and antioxidant enzymes (CAT, GSH-Px, and SOD) in vitro [56]. Moreover, treatment with lycopene metabolites increased also intracellular GSH levels and decreased ROS in cells; they can stimulate ARE transcription system and induce Nrf2-mediated expression of phase II detoxifying/antioxidant enzymes in vitro [57]. Lycopene and β-carotene can inhibit cell proliferation, arrest cell cycle, increase apoptosis of human breast cancer cells in vitro [58].

Curcumin has been shown to attenuate OS and preserve the activity of several antioxidant enzymes by modulating Nrf2 [59] and has also been shown to possess antidiabetic, cholesterol-lowering and anti-inflammatory properties [60].

Resveratrol (RES) is a stilbenoid phenol produced by several plants (most predominant in the skin of berries such as grapes, blueberries, and raspberries) with promising and potent effects on various aspects of human health [61]. RES can inhibit COX-1 and COX-2 and other related proteins involved in cancer progression by lowering angiogenic and metastasis rates in vivo [62,63].

### 2.2. Pro-Oxidant Activity of Nutraceuticals

The term “antioxidant” refers to a wide spectrum of compounds, which can donate electron and neutralize free radicals, resulting in scavenging and prevention of cell injuries. The question remains as to why natural antioxidants do not become toxic while scavenging radicals and converting to free radicals.

The “antioxidant network theory” has been raised because many clinical evaluations of antioxidant supplements to prevent cancer have been unsatisfactory. The reports of the anticancer potential of aromatic compounds found in foods and plants have increased in the recent decades [64]. This chemical class includes polyphenols that are important components of the human diet and that share some typical structural characteristics as the three-membered flavone ring system [64]. Some of these natural substances are present in various pharmaceutical, nutritional, and cosmetic products such as vanillin, which is a plant secondary metabolite and the main constituent of vanilla. Vanillin is a phenolic phenylpropane C6-C1 carbonic structure derivative and acts as an important flavor and aromatic component used worldwide [65]. Vanillin has antitumor potential; a rich diet in such radical scavengers could reduce free radical cancer promotion [66]. In another in vitro study, Apigenin, a natural polyphenolic compound rich in flavones, showed a proapoptotic and antiproliferative properties on cell lines HT-29 (human colorectal adenocarcinoma) and HCT-15 (Dukes’ type C-colorectal adenocarcinoma) models [67]. Other studies have shown promising results on the cytotoxic effect of ginger extracts. Gingerols are a class of phenolic and bioactive compounds used in cancer including breast, cervical, colorectal, leukemia, liver, lung, nasopharyngeal, ovarian, prostate, and retinoblastoma [68]. Recent pharmacological studies have indicated that Gingerols and Shogaoals possess antimicrobial, anticancer, anti-inflammatory, and anti-allergic propensities [69]. Some of these compounds may act as either antioxidants or pro-oxidants to exert protective effects against cancer such as Eugenol [70], an aromatic phenylpropanoid phenol contained in soybeans, coffee, bananas, cinnamon and basil [71,72]. The combinatorial library of polyphenol compounds is widely diversified and includes thousands of different compounds [73]. Polyphenols are found in green and black teas [74,75], coffee [76], fruits and vegetables [77,78], olive oil [79], red and white wines [80], and chocolate [81]. Eugenol has a dual effect on OS: it can act as an antioxidant or prooxidant agent. Moreover, it has antitumor, anti-carcinogenic and cytotoxic properties [70]. Although polyphenols are generally recognized as antioxidants, they also have many other biological activities, such as anti-histamine [82,83], anti-inflammatory, antibacterial and anti-viral activities [84,85], but they can also act as prooxidants, inducing DNA degradation in the presence of metal ions such as copper [86]. Different studies showed that polyphenols involve mobilization of chromatin-bound copper and consequent prooxidant action leading to cell death. Polyphenols with gallol or catechol groups are generally the most potent antioxidants, primarily because of the large iron-binding stability constants for these groups but show even pro-oxidant properties [87]. Due to scavenging ROS and chelating redox metals, herbal polyphenols that are commonly recognized as antioxidants can cause pro-oxidant activity, which may end up with oxidative damage to some biological components [88]. Interestingly, flavonoids and phenolics show prooxidant activity in the presence of transition metals such as iron or copper through chelating redox active metals and reducing them, and then increasing their ability to form free radicals from peroxides [87]. There are some nutraceuticals, such as ascorbic acid (vitamin C), that have both antioxidant and prooxidant properties. The different activities depend on the dosage used [89]. Vitamin C is a CYP inhibitor in vitro [90]. Similarly, quercetin has been shown to have the same activity of CYP inhibition [91].

### 2.3. Combination of Nutraceuticals and Antioxidant Substances

The possible harmful or beneficial effect of an antioxidant compound depends on its concentration, the presence of other antioxidants and the concentration of endogenous antioxidants [92]. In recent years, attention has been focused on the additive and synergistic effects of combinations of phytochemicals [93,94]. Many antioxidants interact with synergic effect known as “sparing effects” [95]. The “synergism concept” between phytochemicals was introduced more than a decade ago by Liu et al. [96,97]. Administration of a mixture of antioxidants is beneficial since they may act synergistically in various phases and may be more effective rather than high amount of a single antioxidant [96]. Using a combination of antioxidants for disease management is preferable, for example, the combination of polyphenols and vitamins have an important role in the prevention of osteoporosis, cardiovascular diseases, cancer, diabetes mellitus, and neurodegenerative diseases [95]. The combined action of phytochemicals exhibits a higher biological effect as compared to the biological effects acquired by the individual phytochemicals alone [98]. In the last decade, it has been shown that phytochemical extracts from fruits and vegetables have potent antioxidant and antiproliferative effects, and it has been proposed that phytochemical combinations from fruits and vegetables were responsible for the potent antioxidant and anticancer activity of these foods [99]. The combination of orange, apple, grape, and blueberry displayed a synergistic effect in terms of antioxidant activity. Fruits and vegetables eaten in the recommended amounts (5–10 servings of fruits and vegetables per day) are safe. Furthermore, among the health benefits from the consumption of fruits and vegetables, there are the reduced risk of developing cancers and cardiovascular disease, and preventive effects on other chronic diseases such as central neurodegenerative diseases, and diabetes [97]. In an in vitro study, Linnewiel-Hermoni et al. observed that different combinations of lycopene, curcumin, vitamin E, and tomato extract, inhibited prostate cancer cell proliferation through a synergistic effect. The cellular system of antioxidant defense was synergistically induced by activation of the Electrophile/Antioxidant Response Element transcription system, which is responsible for induction of several phase-II detoxifying and antioxidant enzymes [100]. Liu and colleagues studied the relationship between different fruit combinations, the synergistic effect and the total antioxidant activity. They observed that plums had the highest antioxidant activity and that combinations of fruit resulted in a greater antioxidant activity which was due to an additive and synergistic effect [97]. In a clinical phase I study it was demonstrated that a regular consumption of grapes (0.15–0.45 kg/day) for 2 weeks reduced mucosal proliferation and Wnt signaling, a pathway constitutively activated in over 85% of colon cancer [101].

During chemotherapy treatment, OS increases, leading to secondary effects that compromise the patient’s quality of life. Hence, it has been proposed supplementation with antioxidants as prophylactic agents in cancer prevention and treatment [102]. Several studies have indicated that anti-cancer efficacy can be enhanced combining polyphenols synergistically with chemically similar or different compounds [103]. In an in vivo and in vitro study, Piao et al. demonstrated anti-cancer effects of a combination of three nutrients, curcumin, resveratrol and epicatechin gallate, on cell viability, apoptosis, clonogenic survival, and tumor growth in HPV-positive head and neck squamous cell carcinoma [104]. Within the group of antioxidants, polyphenols are the most widely studied group of phytochemicals, and in particular resveratrol. RES combined with black tea polyphenol synergistically inhibits skin tumor growth with a consequent decrease of tumor volume [105]. Several phenolic compounds may exert both effects, chemopreventive and anticancer, due to their dual effect on cellular redox regulation. It seems that they promote an antioxidant effect, which leads to the prevention of carcinogenesis in normal cells, and they exert a pro-oxidant effect that favors cell death in cancer cells [106].

Over the last decade, several studies have investigated different combinations of phytochemicals or fruits and vegetables on outcomes of disease prevention, but a clear summary of the most recent scientific findings is lacking. Although natural antioxidants are usually composed of numerous bioactive compounds in different amounts and proportions, it is usually difficult to calculate their safe concentrations, because no such safety tests have been reported. In 2016, based on scientific data, the FDA published the latest Nutrition Facts Label for packaged foods and ordered that the consumers should be better informed of food choices [107]. The only antioxidants listed in the FDA sheets were vitamin C, vitamin E and β-carotene [108]. Despite the numerous health benefits and wide applicability of natural antioxidants, the safety profile of these substances has not been completely ascertained [109]. However, a maintenance dose of antioxidants, before or after the chemotherapy could be recommended.

## 3. Effects of Nutraceuticals on MicroRNAs

**Figure 2:**
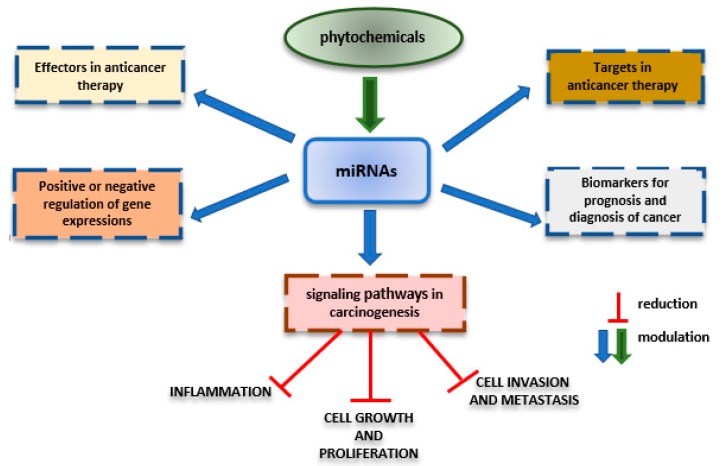
Phytochemicals and miRNAs involved in carcinogenesis process.

Bioactive phytochemicals influence several physiological processes, such as gene expression, cell cycle regulation, cell proliferation, cell migration, etc., resulting in cancer prevention [110]. They could modulate non-coding RNAs (ncRNAs), up-regulate tumor suppressive microRNAs (miRNAs), and down-regulate oncogenic miRNAs inhibiting cancer cell growth and cancer stem cell self-renewal [111]. Nutraceuticals could modulate miRNAs expression and consequently might influence cellular responses in health or diseases conditions, including cancer (Figure 2) [112]. miRNAs are the most abundant non-coding regulatory RNA molecules in species ranging from human to viruses, and they have been shown to have key roles in the regulation of gene expression [113]. miRNAs are engaged in the control of a large variety of physiologic processes, as development, differentiation, neuronal asymmetry, metabolism, stem cell biology, proliferation and programmed cell death, and they may drive, potentiate or repress oncogenesis [114]. A key target of the effects of natural products may be the regulation of miRNAs expression, which results in cell death or prevents aging, diabetes, cardiovascular and other diseases [14]. Numerous studies have reported a dysregulated expression of miRNAs in thyroid cancer evaluating their diagnostic and prognostic role [115]. Allegri et al. demonstrated, in vitro, that treatments with four different nutraceuticals (curcumin, RES, genistein and EGCG), induced heterogeneous effects on miRNA levels in thyroid cancer cell line; moreover, strong and positive effects was observed only after curcumin treatment [116]. 

Literature data have described a relationship between phytochemicals, such as RES and curcumin, and modulation of miRNAs. For instance, Lelli et al. demonstrated the anti-cancer activity of curcumin in vitro by inducing modulation of several miRNAs in different human cancer cells [117]. Bosutti et al. tested the effects of RES, curcumin and genistein on prostate adenocarcinoma in vitro. Genistein is arousing interest due to its preventing properties in prostate adenocarcinoma [112]. In vitro it up-regulates mRNA expression of some tumor suppressor genes, including glutathione S-transferase P1 (GSTP1) and ephrin B2, through the demethylation of their promoter regions and it produces the inhibition of pro-proliferating factors such as mouse double minute 2 homolog (MDM2), Akt and NF-kB [118]. Moreover, genistein has also been shown to either down-regulate or up-regulate miRNAs genes, and in vitro studies in prostate carcinoma cells indicate that genistein may also directly modulate the expression of miR-1260b, miR-222, miR-221, miRNA-151, miR-1296 and miR-574-3p, where miR-1260b is an oncomiRNA highly expressed in prostate cancer tissues, wherein it inhibits apoptosis and promotes cell growth and invasion [119]. Epigenetic data demonstrate that RES, similarly to genistein, can reduce promoter methylation of some genes, including miRNA genes. In particular, microarray analysis in prostate cancer cells, LNCaP and DU145 cells, has revealed that RES either reduces or increases the levels of many miRNAs associated with prostate tumor; the main oncomiRNAs, negatively modulated by RES, commonly inhibit the expression of phosphatase and tensin homologue deleted on chromosome 10 (PTEN), a tumor suppressor that is down-regulated in several types of cancer [120]. In BC, RES up-regulated tumor-suppressive miRNAs (such as miR-16, miR-141, miR-143, and others) in MDA-MB-231 cell lines and showed anticancer effects against BC stem cells in vivo [121]. Despite numerous in vitro studies concerning the effects of plant-derived compounds on levels of miRNAs in BC cell lines, there is a lack of in vivo studies validating these findings [111]. Curcumin, in an in vitro study on murine prostate cancer cells, elicited demethylation of the promoter of *Nrf2* gene, a master regulator of detoxifying/antioxidant enzymes, leading to the re-expression of Nrf2 and NAD(P)H dehydrogenase, and quinine-1(NQO-1), key players in the antioxidant and stress responses [122]. However, more preclinical and clinical studies are needed to elucidate how natural compounds influence the process of carcinogenesis by influencing the levels of miRNAs.

### Nutraceuticals’ Impact on Epigenetic Phenomena in Cancer

In the last decade, studies have focused on certain groups of botanic components with bioactive properties in influencing epigenetic processes. These bioactive compounds can be consumed as part of the human diet, and are collectively referred to as the “epigenetic diet” [123]. Some dietary components with properties that influence epigenetic processes are believed to have preventive effects on many human diseases, such as cancer. Numerous dietary and natural phytochemicals (such as catechins, curcumin, genistein, quercetin and resveratrol, others) exhibit potent anti-tumor activities through the reversion of epigenetic alterations associated with oncogenes activation and tumor suppressor genes inactivation; moreover, they can modulate the mammalian epigenome through regulation of mechanisms and proteins responsible for chromatin remodeling [124]. Human epidemiological and animal studies have shown that early consumption of certain epigenetic diets may lead to an epigenetic modulation and they can reduce the risk of the development of certain diseases [125]. Experimental studies have dealt with modifying the activity of proteins and ncRNAs by plant-derived compounds, such as phytochemicals, which are involved in modulating epigenetic mechanisms and in shaping the epigenome, showing that they could have great importance in pharmacogenomics in the near future [126]. It has been shown that they participate in DNA methylation, histone modifications, and post-transcriptional regulation of genes through affecting ncRNAs, especially miRNAs and long ncRNAs [127]. These dietary compounds, if proper dietary guidelines are followed, can have effects on prevention of various human diseases such as cancer and diabetes. Many human foods contain epigenetic dietary components, such as genistein, a natural isoflavone in soybean products, sulforaphane, an isothiocyanate found in broccoli sprouts or cabbage, and EGCG, the major polyphenol in green tea, which have been found to be associated with a lower risk of developing many common cancers [128]. Dietary compounds are the primary components that regulate gene expression by epigenetic mechanisms such as DNA methylation and histone modification through histone acetyl transferases (HATs) and histone deacetylases (HDACs). Epigenome modifications may be altered by long-term consumption of bioactive compounds, this can contribute to the development of nutritional programs to prevent and treat metabolic diseases. [110]. These modifications are a stable and heritable component of epigenetic regulation that represents an important memory mechanism during embryogenesis [129]. DNA methylation patterns are maintained by at least three independent DNA methyltransferases (DNMTs), DNMT1, DNMT3a, and DNMT3b that are required for cellular differentiation during early embryonic development; thus, an appropriate exposure to epigenetic modulators from the diet that target DNA methylation may lead to beneficial intervention of early epigenetic reprogramming and disease prevention in later life [127]. Usually, cancer treatments involve the use of chemo-radio therapeutic agents, kinase inhibitors, personalized antibodies, and compounds that stimulate the immune system. Demethylating drugs and histone deacetylase inhibitors can modify gene expressions by reversing the aberrant epigenetic alterations acquired during tumorigenesis [130]. Recent studies have reported that phytochemicals may represent an alternative therapeutic option for cancer treatment and that dietary supplements and natural compounds may restore the normal epigenetic marks that are altered during carcinogenesis [123]. In cancer, the most studied phytochemicals are EGCG, quercetin, RES, curcumin, and sulphorane, which block the development and progression of tumors by targeting key signaling transducers such as the modulation of epigenetic machinery (regulation of DNMTs and HDACs activities) [131,132]. Khan et al. showed, through studies of molecular modeling, that EGCG directly binds to the enzymatic substrates of DNMT3b and HDAC1, leading to their inhibition and the reactivation of tumor suppressor genes such as retinoic acid receptor β, cadherin1 and death-associated protein kinase-1 [133]. In another study, Lee Y.H. et al. reported that, in vitro, EGCG repressed the hormone responsiveness of androgen receptor by reducing the acetylation of this receptor, leading to decreased cell proliferation and promoting cell death in LNCaP prostate cancer cell line. In cervical and skin cancers, EGCG is a potential epigenetic modifier of DNMTs and HDACs and restores epigenetically silenced genes. In skin cancer cells, EGCG decreased the DNMT1, DNMT3a, and DNMT3b protein levels and modulated the HDAC activities allowing the transcriptional activation of tumor suppressor genes such as p16 INK4a and Cip1/p21 [134]. Another phenolic component, curcumin, is widely used in China and India for medicinal purposes and for its antioxidant, anti-inflammatory, anti-proliferative, anti-angiogenic, and anti-cancer properties [135]. It has been considered an excellent non-toxic hypomethylating agent for BC therapy [136]. For example, curcumin inhibited DNMT1 expression and restored the function of RASSF1A by promoter hypomethylation in estrogen positive MCF-7 breast cancer cell line and decreased the cell proliferation and breast tumors growth in vivo [137]. When A549 lung cancer cells were implanted in nude mice and treated with curcumin, it was observed that tumor growth decreased, and this effect was mediated by increasing of RARβ and decreasing of DNMT3b expression [138]. On the other hand, curcumin induced histone hypoacetylation and apoptosis associated with PARP activity in brain cancer cells and impeded differentiation of astrocytes and promoted neural differentiation associated with hypoacetylation of H3 and H4 [139]. In in vivo and ex vivo studies of different cell line models of acute myeloid leukemia, curcumin down-regulated the expression of DNMT1 and restored p15INK4b expression by hypomethylation of its promoter inducing cell cycle arrest at G1 phase and apoptosis [140]. Curcumin inhibits JNK signaling and represses H3K4me3 epigenetic mark promoting apoptosis of LNCaP prostate cancer cells in vivo [141]. RES is a phytoalexin that provides therapeutics effects in different types of cancer regulating biological functions such as cell proliferation, cell division, apoptosis, angiogenesis and metastasis [142]. RES promotes acetylation and reactivation of PTEN by inhibition of the MTA1/HDAC complex, as well as inhibition of the Akt pathway in vivo. Thus, MTA1/HDAC complex is a negative regulator of PTEN, which promotes tumor cell survival and progression of prostate cancer [143]. Above all, the role of phytochemicals in preventing the occurrence/recurrence of cancer is still under debate, as well as their potential in nutritional intervention for modulating the functions of epigenetic mechanisms.

## 4. Nutraceuticals: Prevention, Cure and Chemotherapy

Nowadays, thanks to modern cancer treatments, the survival rate of patients has significantly improved. This enhancement of survival rate shows progress in early stage diagnosis and of the use of combination chemotherapy. Clinical studies have shown a synergistic effect of natural antioxidants with certain chemotherapeutic agents. A randomized trial conducted on 100 patients with BC diagnosis, showed an increased response rates in patients receiving vitamin A supplements in addition to either doxorubicin or cyclophosphamide [144].

Drisko et al. conducted a small prospective study with two patients diagnosed with advanced epithelial ovarian. Both patients received carboplatinum/paclitaxel. While patient 1 was administered a combination of oral antioxidants (vitamins C, E, b-carotene, coenzyme Q-10, and a multivitamin/mineral) prior to carboplatinum, patient 2 also received the chemotherapy combination, the antioxidant combination and the parenteral ascorbic acid but did not receive the consolidation paclitaxel. The results showed synergy between antioxidants and chemotherapy when administered in combination, leading to remission in both patients [145]. Zhao et al. demonstrated that low doses of Vitamin C and Decitabine have synergistic effects on proliferation, apoptosis, TET2 expression and activity when compared to drug-alone treatment in HL60 and NB4, both in cell lines in vitro and in clinic study. Safety evaluations showed that patients who received an intravenous Vitamin C with Decitabine (A-DCAG) regimen had a higher complete remission rate than those who received the Decitabine regimen (DCAG) after one cycle of chemotherapy [146]. Zeichner and colleagues found that women receiving a little over 10,000 IU/week (≈1500 IU/d) of vitamin D during chemotherapy for BC had a statistically significant improved disease-free survival when compared patients undergoing the same treatment but not taking vitamin D supplements [147].

In addition to the unstable position of nutraceuticals in cancer therapy, another argument still open regards their capacity to prevent occurrence of cancer. Do nutraceuticals protect cancer cells from the effect of chemotherapy? The answer is complicated by the stage of cancer progression and by their dual role as antioxidant or prooxidant [148].

Chronic diseases are the leading causes of death and disability. Although many studies have found protective roles for nutraceutical phytochemicals in chronic diseases, other studies have found some discordances. Many potential drug-nutrient interactions can affect cancer treatment [149]. In the last decade, people have paid close attention to the role of nutraceuticals in tumor prevention and cancer therapy because of their extensive sources, low cytotoxicity and safe consumption [150]. At present, oncologists suggest to a lot of patients to avoid dietary supplements with antioxidant capacity such as vitamins during cancer therapies. This kind of warning is not easy to realise, because almost all foods (including fruits, vegetables, beans, and nuts) consist of antioxidants and vitamins, or similar substances [149]. High doses of phytochemical extracts in the daily diet may not be safe, or may have toxic effects. It is necessary to differentiate the physiologic (nutritional) dose from the pharmacological dose.

Regarding the use of phytochemicals with antioxidant properties during radiation and chemotherapy, two opposite hypotheses have been proposed. One supports supplements with high doses of multiple dietary antioxidants, such as vitamins C and E, and carotenoids, may improve the efficacy of radiation or chemotherapy by increasing tumor response and decreasing toxicity. The other hypothesis suggests that antioxidants (dietary or endogenously made), should not be used during radiation therapy, because they would protect cancer cells against radiation damage [150]. The critical point in the contemporaneous consumption of antioxidants in chemotherapy is due to the lack of clinical evidence; scientific evidence on this topic is not strongly “for” or “against” taking antioxidant supplements during cancer treatment.

Chemotherapy is used primarily to treat systemic diseases rather than localized lesions. In chemotherapy treatment, tumor cells are destroyed with antineoplastic agents that interfere with cellular function (including replication). This drug treatment causes lethal injury to DNA, which leads to further malignant cell death via apoptosis [151]. Chemoprevention is the use of non-toxic chemical substances to interfere with neoplastic development [8]. Chemopreventive phytochemicals present in plant-derived foods are potential modifiers of signal transduction pathways mediated by NF-kB that lead to cancer development [151]. NF-kB is a transcription factor that alters the genes of cell survival, cell adhesion, inflammation, differentiation, and growth, promoting cancer development. Among the chemopreventive phytochemicals known to suppress carcinogenesis by blocking NF–kB activation process, there are curcumin (turmeric), catechins (tea), caffeic acid, capsaicin (red chilli), resveratrol (red grapes, peanuts and berries), lycopene (tomato), Beta-carotenes (carrots), 6-gingerol (ginger), ursolic acid (rosemary), ellagic acid (pomegranate), ajoene, allicin, diallyl sulfide (garlic), and many others [149]. Dietary phytochemicals have anti-inflammatory and anti-oxidative properties that can contribute in chemopreventive activities (Table 1).

**Figure 3:**
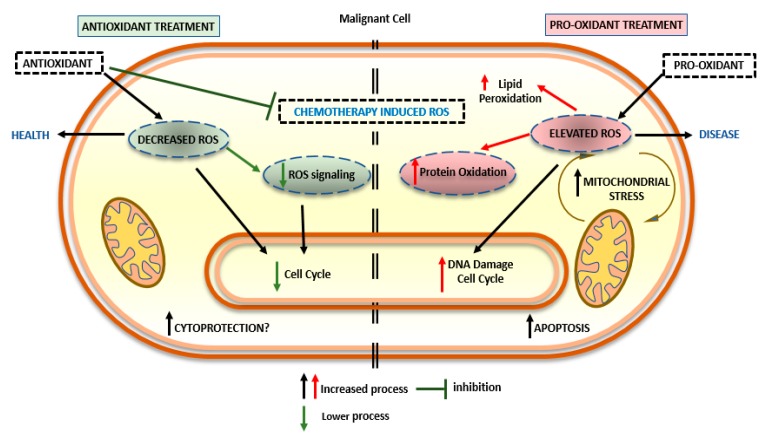
Antioxidant treatment versus pro-oxidant treatment during chemotherapy.

Certain chemotherapeutic agents involve the generation of free radicals to cause cellular damage and necrosis of malignant cells, but these ROS often can cause side effects that remains during all the period of the treatment [152]. Nutraceuticals can antagonize cancer therapies by interfering with the late-stage cancer and they possibly can alter the course of metastatic spread of cancer (Figure 3) [153]. It has been documented, for example, that garlic has anti-coagulant properties and also ginger and ginko supplements can interact with warfarin. Garlic and its organosulfides may interact to suppress the function of cytochrome P4502E1 involved in the metabolism of analgesic acetaminophen [154]. This is a reason for doctors recommending the disuse of non-steroidal anti-inflammatory drugs prior to surgery [155,156]. To kill cancer cells, chemotherapy drugs cause high levels of OS, and this mechanism might interfere with the effectiveness of chemotherapy [151]. The main point is that OS slows the process of cell replication, but chemotherapy kills cancer cells exactly during cell replication thus, slowing cell replication means lowering chemotherapy efficacy [155]. To decrease OS and make the chemotherapy treatment more effective, one approach is the addition of certain antioxidants, at specific dosages, to the patient’s diet [157,158]. Some antioxidants can reduce aldehyde generation during chemotherapy-induced oxidative stress in order to enhance the antioxidant activity of cancer chemotherapy [159].

The controversial role and the different effect of phytochemical nutraceuticals with antioxidant activity in cancer therapies changes based upon the different dosage used: low doses of antioxidants for a preventive treatment and high doses of antioxidants for therapeutic treatment [160]. Data have shown that the preventive dose protects tumor and normal cells, but the therapeutic dose inhibits tumor cell growth but not that of normal cells. Most preparatory clinical trials have declared that antioxidants do not significantly reduce the effect of chemotherapies [161]. Numerous articles have focused their attention to understand whether the administration of antioxidant supplements during chemotherapy could protect normal cells without interfering with tumor control. Other recent reviews have shown that antioxidants, when given concurrently, protect normal tissues, and increase patient’s survival and therapeutic responses without interfering with chemotherapy [162,163]. One of the key processes in cancer development is angiogenesis which is stimulated by formation of new blood vessels from endothelial cells with sustained oxygen and nutrient supply.

Angio-prevention is a new concept that analyzes the mechanisms on how and why chemopreventive agents could exert their antiangiogenic effects. Angio-prevention aims to control tumor growth through the inclusion and use of both natural and synthetic chemopreventive agents [164].

Polyphenols such as curcumin, resveratrol, and genistein, possess strong antiangiogenic properties. They can inhibit the formation of new blood vessels, showing antioxidant properties that directly scavenge ROS [165]. Anthocyanidin and ellagic acid (a natural polyphenol found in several fruits and nuts) are known to inhibit, in vivo and in vitro, vascular endothelial growth factor (VEGF) and platelet-derived growth factor (PDGF) receptors that play a complementary role in angiogenesis [166]. This combined inhibition of VEGF and PDGF leads to inhibition of angiogenesis process in both in vitro and in vivo assays [167], suggesting that the antiangiogenic property of phytochemicals plays a crucial role in their chemopreventive activity by preventing the formation of new blood vessel that is necessary for cancer development.

Cancer patients in their late stage show an impaired immune-physiological condition, including decreased activity of natural killer (NK) cells and cytokine production. In this late stage of cancer, nutraceuticals can considerably induce production of cytokines such as tumor necrosis factor, interferons, interleukins, and potentially activate natural killer cells, T lymphocytes and macrophages [168]. In this study, Chen and colleagues evaluated the effects of Ganoderma lucidum (Lingzhi), a medicinal mushroom widely used by Asian people, on selected immune functions in patients with advanced colorectal cancer. The immune-modulating effect was studied in 41 assessable cancer patients after treatment (three times daily for 12 weeks) with the polysaccharide fraction from G. lucidum. In this study, this herb tended to increase mitogenic reactivity to phytohemagglutinin, counts of CD3, CD4, CD8 and CD56 lymphocytes, plasma concentrations of IL-2, IL-6 and interferon-γ, and NK activity, whereas plasma concentrations of IL-1 and tumor necrosis factor (TNF)-α decreased but statistical significance was not observed when a comparison was conducted between baseline and those values after a 12-week treatment. For example, the changes of IL-1 were correlated with those for IL-6, IFN-γ, CD3, CD4, CD8 and NK activity (*p* < 0.05) and IL-2 changes were correlated with those for IL-6, CD8 and NK activity. Results from the present study suggest that G. lucidum may act as a host defense potentiator in patients with advanced colorectal cancer [168].

There are many therapeutic approaches that recommend phytoestrogens and soy isoflavones (such as genistein, daidzein, biochanin) that aim to modulate the expression of IL-6 gene to prevent cancer progression [169]. Soy isoflavones are considered to be a potential alternative to the synthetic selective estrogen receptor modulators (SERMs), which are currently applied in hormone replacement therapy (HRT). As phytochemicals integrate hormonal ligand activities and interference with signaling cascades, they may have applications in cancer chemoprevention and/or NF-κB-related inflammatory disorders as well. Excessive IL-6 production promotes tumorigenesis (in breast, prostate, lung, colon, ovarian cancer), in this respect, to prevent cancer progression, a pharmacological modulation of IL-6 gene expression levels may have therapeutic benefits in human. The transcription factor NF-κB is a key player in IL-6 gene expression which transcriptional activity is regulated at multiple levels. Dijsselbloem et al. and other groups obtained convincing evidence indicating that isoflavones have a potential NF-κB-inhibitory activity and/or inhibitory effects on targets of the inflammatory/apoptotic cascade [169,170].

Greenlee and colleagues showed that nutraceuticals may provide some benefits when combined with certain types of chemotherapy. Frequent use of vitamin C and vitamin E was associated with decreased risk of BC recurrence, vitamin E use was associated with decreased risk of all-cause of mortality, but conversely, frequent use of combination carotenoids was associated with increased risk of death from BC and all-cause mortality [171,172]. The study of Grenlee and colleagues showed that the effects of antioxidant supplement use, after diagnosis, differed by type of antioxidant. This study was conducted in 2264 women, and associations between antioxidant use in BC diagnosis and BC outcomes were examined in the Life After Cancer Epidemiology cohort. Starting from the hypothesis that different forms of antioxidant supplements would have differing effects on outcomes, a protective association was observed between use of vitamin C and vitamin E and BC recurrence and death from all causes. Data showed that frequent use of combination carotenoids in the period following diagnosis was associated with an increased risk of death from BC and all causes, but not BC recurrence, and it was also observed that frequent use of vitamins C and E after diagnosis was associated with reduced risk of all-cause mortality, death from BC, and BC recurrence [172].

Vitamin supplement in the first 6 months after BC diagnosis may be associated with reduced risk of mortality and cancer recurrence [173]. Nechuta and colleagues evaluated the association of vitamin supplement use in the first six-months after BC diagnosis and during cancer treatment with total mortality and recurrence. The study was conducted in a population-based prospective cohort of 4877 women diagnosed with invasive BC. Women who used antioxidants (vitamin E, vitamin C, multivitamins) for a short period after the diagnosis had 18% reduced mortality risk and 22% reduced recurrence risk, independent of multiple lifestyle factors, clinical prognostic factors, and socio-demographics [173].

Vitamin E is also known to induce apoptosis in experimental tumor lines and has shown capacity to increase the efficiency of chemotherapy [174]. In another study, Vitamin E was shown to decrease chemotherapy-mediated toxicity and with omega-3 fatty acid increase survival time in terminal cancer patients [175].

Nutraceutical industries have increased the production of a lot of phytochemicals containing nutraceuticals because of the increased use of phytochemicals in chemoprevention, but the weak point of phytochemical use in cancer is that research is conducted in vitro.

## 5. Discussion

Cancer is a chronic multifactorial disease, and several factors contribute to its growth, one of which is an inadequate diet. Many recent studies have shown that there is an association between diet and cancer and that nutraceuticals do exert important biological effects on human cells. In many cases, the development of cancer is associated with ROS generation. Free radicals can play a dual role as accelerator or inhibitor of carcinogenesis under different conditions [148]. Therefore, the big question is “do I take or avoid nutraceuticals for cancer prevention and during chemotherapy?” (Figure 3). Nutraceuticals are defined as nutrients or functional foods or spices (plants) with pharmaceutical activity [1]. They are food supplements that have been reported to maintain healthy bodies and have pleiotropic effects in metabolic pathways to preserve health conditions. Hippocrates enforced the old use of herbs, anticipating a novel consideration of food being related to health maintenance. The choice of different nutraceutical supplements can normalize body functions (even under disease conditions), prevent diseases, and strengthen the immune system. In the modern era, bioactive foods have been used as anticancer, antimicrobial, immunity booster, antidiabetic, and gastro-protective agents. The use of nutraceuticals in patients with cancer has been discussed for a long time without any definitive conclusion. More emphasis needs to be placed on understanding the benefits of nutraceuticals in the prevention or in the treatment of cancer. Some nutraceuticals have benefits in arresting cancer growth and progression by inhibiting stem cell potential migration and invasion [14]. Bioactive compounds can improve the effect of chemotherapy by rendering cells more sensitive to the different treatments and by lowering the effect of the chemotherapy. The mechanistic aspects of nutraceuticals have not been fully elucidated: (i) they could affect epigenetics by improving or down-regulating DNA methylation or protein acetylation; (ii) they could act by activating specific miRNAs; (iii) they could block inflammatory pathways by inhibiting NF-κB and subsequently down-regulating b-cell lymphoma-2 (Bcl-2), ciyclyn D1, metallo-proteinases-9 (MMP-9), angiogenic factors and IL-6 pathways [20,95,110]. Moreover, bioactive nutraceuticals could inhibit electron transport chains and activate AMPK by increasing its phosphorylation. Nutraceutical bioactive food has been reported to have antioxidant activity with the opposite effects: it could increase antioxidant activity, having a beneficial effect by arresting the spread of cancer while at the same time rendering the cells resistant to chemotherapy that uses ROS production as a mechanism. Furthermore, natural compounds can improve chemotherapy at different dosages by explicating a prooxidant activity resulting in a synergistic effect with chemotherapeutic agents (Figure 3).

## 6. Conclusions

**Figure 4:**
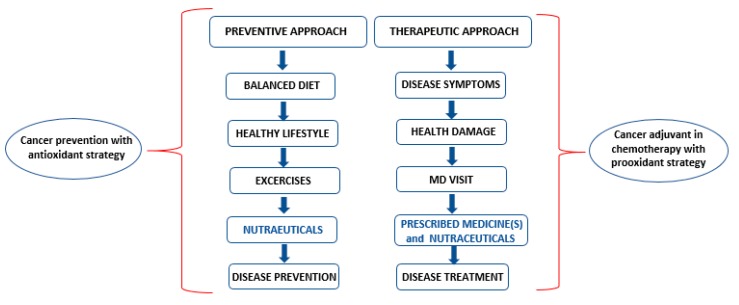
Different approaches in cancer prevention and chemotherapy treatment.

A lot of patients, after cancer diagnosis, turn to nutraceuticals in the belief that this dietary supplementation could have good benefits. Certain plant-based supplements may antagonize and alter the metabolism of therapeutic agents, influencing their effectiveness. Some natural compounds, alone or in combination with diet, can provide some benefits for chemoprevention. These natural agents have inhibited, at least experimentally, the initial stages of carcinogenesis, cancer spread and metastasis, but this area of research requires further in-depth study. This review aims to suggest that the introduction into the diet of nutraceuticals with antioxidant activity can help the prevention of cancer, lowering chemotherapy efficacy, while nutraceuticals with prooxidant activity can interfere with chemotherapy, increasing its effectiveness (Figure 4). This is one of the reasons why patients should weigh the risks and benefits of nutraceuticals during and after chemotherapy treatment.

## Figures and Tables

**Table 1:** Experimental studies of different nutraceuticals alone and combined with chemotherapy.

Nutraceuticals	Doses	Model	Effects of Nutraceutical Alone	Combined Effect with Chemotherapy	Ref.
Ascorbic acid (vitamin C)	Vitamin C (0–20 mM) + various doses of chemotherapic drugs	In vitro	increased ROS and anti-tumorigenic effect with high doses of vit C in cancer cells without meaningful toxicities to normal cells	high-doses vitamin C inhibited cancer cells proliferation, increased apoptosis in BC cells; additional inhibitory effect on cells growth	[176]
Berberine	BBR from 2.5∼320 µM doxorubicin different concentrations	In vivo and in vitro	AMPK activator, activated caspase; PARP-1 cleavage; Cytochrome c release; cell cycle arrest	BBR sensitized drug-resistant BC to chemotherapy; directly induced apoptosis through the dose-orchestrated AMPK signaling pathway	[177]
Carotenoids, lycopene	0.5–10 μM	In vitro	cell proliferation inhibition, cell cycle arrest, increased apoptosis of cancer cells	Protective effects against cisplatin-induced nephrotoxicity and doxorubicin-induced cardiotoxicity	[178,179]
Epigallocatechin-3-gallate (EGCG) derivatives	ECGC derivatives + cisplatin (2mg/kg per 2 days)	In vivo (mouse) and in vitro	cell-cycle arrest, inducted apoptosis and ROS, inhibited NF-ĸB, HER-2/neu, (IGF-1)-mediated and EGF-mediated signaling pathways, inhibited proteasome activity, iNOS, MMPs, VEGF, AP-1, MAPKs and COX-2 expression	EGCG derivatives inhibited cell viability and colony formation, caused cell cycle redistribution, induced apoptosis. Co-treatment enhanced apoptosis rate; reduced tumor growth.	[180,181]
Curcumin	20 μM curcumin10nM docetaxel	In vitro	cell proliferation inhibition, anti-invasive activity, angiogenesis inhibition; Nrf2 enzymes activation; promoted tumor suppressor p53 and TGF-β and COX-2 reduction	curcumin enhanced efficacy of docetaxel, inducted apoptosis, inhibited proliferation, down-regulated NF-κB, COX-2, RTKs, and kinases PI3K and phospho-AKT by combined treatment	[182]
Eugenol	1 μM eugenol + 30 μM cisplatin (vitro) 2 mg/kg cisplatin + 50 mg/kg eugenol (in vivo)	In vitro and in vivo (mouse)	growth and proliferation inhibition, induced apoptosis through targeting the E2F1/surviving pathway	Co-treatment significantly increased cytotoxic and pro-apoptotic effects, eugenol potentiated cisplatin inhibition of the NF-κB signaling pathway. Down-regulation of the IL-6 and IL-8 cytokines; inhibited epithelial-to-mesenchymal transition and stemness markers in tumor xenografts.	[183,184]
Genistein	1 μM genistein, 10 μM cisplatin, 10 nM paclitaxel, 10 μM tamoxifen	In vitro	cell cycle arrest, improved mitochondrial functionality, regulated OS, uncoupling proteins, antioxidant enzymes and sirtuin, enhanced effects of anticancer drugs	Co-treatment increased cell viability and antioxidant protein levels, decreased ROS, decreased autophagy and apoptosis	[185]
Gingerol	10 μM gingerol or 300 μM gingerol+ 2 μg/mL cisplatin	In vitro	inhibited proliferation and metastasis, cell cycle arrest through inactivation of Akt and p38MAPK activity, suppressed epidermal growth factor receptor expression	Co-treatment inhibited cell viability, enhanced cell cycle arrest at G1 phase; inhibited cell migration and invasion ability; decreased cyclin D1, cyclin A2, MMP-9, p-PI3K, AKT, and p-AKT protein expressions and increased P21 and P27 mRNA levels.	[186,187]
Quercetin	20 μM quercitin + 40 μM metformin	In vivo (nude mice) in vitro	induced apoptosis, induced ER stress, activated pSTAT3/Bcl2 axis, induced protective autophagy	Co-treatment synergistically inhibited growth, migration and invasion of cancer cells, strongly inhibited the VEGF/Akt/PI3K pathway; the increased apoptosis was caspase-dependent and by down-regulation of Bcl-2 family members	[188]
Resveratrol	Differential concentrations	In vivo (rats)	Inhibited CYPA1 drug metabolism and COX activity. Suppressed TNF-α and IL-17. Influenced fatty acids oxidation, mitochondrial biogenesis, respiration, gluconeogenesis	RES prevented bone loss from MTX chemotherapy–induced and bone marrow adiposity; bone-protective properties (pro-osteogenic, antiresorptive, and antiadipogenic)	[17,189]

## Abbreviations

AC	Caffeic acid
AMPK	Adenosine monophosphate protein kinase
BBR	berberine
BC	breast cancer
Bcl-2	b-cell lymphoma-2
CAT	catalase
COX-1	Cyclooxygenase 1
COX-2	Cyclooxigenase 2
CYP	Cytochrome P450
DNMT	DNA methylation
EGC	epicatechin gallate
GPx	glutathione peroxidase
HDAC	DNA histone deacetylases
IL-2	interleukin 2
IL-6	interleukin 6
IL-17	interleukin 17
MAPK	mitogen-activated protein kinase
Met	metformin
miRNA	microRNA
MMP-9	metallo-proteinases-9
NF-κB	nuclear factor kappa-light-chain-enhancer of activated B cells
NK	natural killer
NOS	nitric oxide synthase
NRF2	nuclear factor (erythroid-derived 2)-like 2
OS	oxidative stress
RES	resveratrol
ROS	reactive oxygen species
RONS	reactive oxygen nitrogen species
RNS	reactive nitrogen species
SOD	superoxide dismutase
Trx	thioredoxin
TNF-α	tumor necrosis factor-alpha
PDGF	platelet-derived growth factor
VEGF	vascular endothelial growth factor
